# Increased risk of health professionals to feel traumatized during the COVID-19 pandemic

**DOI:** 10.1038/s41598-021-97783-6

**Published:** 2021-09-14

**Authors:** André Luiz Carvalho Braule Pinto, Alexandre Luiz Oliveira Serpa, Jonas Jardim de Paula, Danielle Souza Costa, Kelly Robis, Alexandre Paim Diaz, Rui Mateus Joaquim, Antônio Geraldo da Silva, Débora Marques de Miranda, Leandro Fernandes Malloy-Diniz

**Affiliations:** 1 SAMBE - Instituto de Saúde Mental Baseada em Evidências, Rio de Janeiro, Brazil; 2 Post Graduation, Department in Molecular Medicine, Universidade Federal de Minas Gerais, Belo Horizonte, Brazil; 3 Department of Psychology, Faculdade de Ciências Médicas de Minas Gerais, Belo Horizonte, Brazil; 4 SCNLab, Universidade Presbiteriana Mackenzie, São Paulo, Brazil; 5 The University of Texas Health Science Center at Houston (UTHealth), Houston, USA; 6 Associação Brasileira de Psiquiatria, São Paulo, Brazil; 7 Faculdade de Medicina da Universidade do Porto, Portugal, Brazil; 8 Department of Psychiatry, Universidade Federal de Minas Gerais, Belo Horizonte, Brazil; 9 Present Address: Department of Pediatrics, Universidade Federal de Minas Gerais, Av. Prof. Alfredo Balena, 190/Sl. 114 - Santa Efigênia, Belo Horizonte, MG Brazil

**Keywords:** Risk factors, Fatigue

## Abstract

Health professionals may be a vulnerable group to posttraumatic stress symptoms (PTSS) during the Coronavirus disease 2019 (COVID-19) pandemic. To investigate how health professionals who experienced a traumatic event are expressing PTSS and factors related to risk for higher PTSS symptomatology can inform how health professionals are facing their role in this crisis. This was an Internet cross-sectional survey. Participants were 49,767 Brazilian health professionals who have ever faced a traumatic event, which was about 25.9% of an initial sample of health professionals. PTSS symptoms were assessed using the Impact of Event Scale-Revised (IES-R) and latent profile analysis (LPA) explored subpopulations within participants based on their scores. Distinct profiles were compared for psychological distress (e.g., depression and anxiety) and quality of life. Multinomial logistic regression analysis was conducted to investigate the relationship between IES-R profiles and COVID-19 related experiences, thoughts, and perceptions. A two-profile model was the most appropriate for the IES-R data pointing out a group with a high level of PTSS (named high-PTSS; n = 10,401, 20.9%) and another expressing a low level of symptoms (named low-PTSS; n = 39,366, 79.1%). The high-PTSS profile demonstrated worse psychological scores (global psychological distress, somatization, depression, and anxiety) and worse quality of life (physical, psychological, social, and environmental) with moderate magnitudes. Small but significant predictors of the high-PTSS profile included sociodemographic characteristics and COVID-19 related experiences, thoughts, and perceptions. Most individuals who experienced a traumatic event were not in the high-PTSS profile. For those who were, however, psychological and quality of life measures were much worse. During the initial phase of the COVID-19 pandemic, several characteristics emerged as risks to report trauma.

## Introduction

Currently, the world is living with a rapidly evolving viral infection, the Coronavirus disease 2019 (COVID-19) pandemic that is drastically altering people's lives bringing with it unprecedented hazards to mental health^1^. Disasters such as a pandemic, when followed by extended periods of high cumulative adversity associated with breakdown of infrastructure, family stress, loss of life and property, ongoing economic consequences, and other factors that hinders overall recovery, pose a much greater risk of mental health problems surgency and persistence^2,3^.

Catastrophic events or disasters, being natural or technological^4^, frequently extrapolate or overload local capacity, demanding external assistance from national or international levels; they are unexpected events that cause significant damage, destruction, and human suffering^4,5^. Such scenery lays down caution about stress-related disorders, which is the case for posttraumatic stress disorder (PTSD), a debilitating, and often chronic, psychiatric disorder^6^.

Posttraumatic stress symptoms (PTSS) include intrusive thoughts, flashbacks, nightmares, sleep disturbances, avoidance of people or circumstances related with the event, emotional numbing, physiological hyperarousal, and attentional and memory changes after traumatic events^7^. In turn, posttraumatic stress disorder (PTSD) must be associated with functional impairment and persist for at least a month after the traumatic event^8^. Both acute and persistent chronic stress modulate fear regulation, resulting in deficits in fear extinction and amplified fear generalization even in a safe environment, which can lead to brain connectivity and circuits’ changes and to functional impairment^9,10^.

Events that are threatening to life or disrupt one’s physical integrity and are out of the usual range of life experiences can be traumatic. Then, it is not unexpected the rise of psychological distress and trauma in the present pandemic^1^. COVID-19 disruptive and disorganizing characteristics may imply a trauma for many experiencing the pandemic. It was an unexpected and dreadful event for every affected country, though differences in handling the pandemic might incur different outcomes. As in previous disasters, the aftermath of the pandemic can imply loss of activities and occupations, social and economic instability, disturbance of the feeling of belonging and promotion of migration^11,12^.

PTSS/PTSD prevalence and specifics depending on groups, as in the case of health professionals, are already assuming characteristics that must be studied. For example, a study from China concerning the current pandemic confirmed a greater incidence of PTSS in women and demonstrated a higher risk of PTSS for health professionals and people with sleep problems^13^. Previous viral outbreaks have taught us about risk factors for PTSS in such events. In the SARS outbreak in China, a study^14^ demonstrated that people in quarantine, those working in high-risk conditions, or who had close friends or relatives infected were two to three times more likely to develop high levels of PTSS than those not exposed to the virus.

The 12-month prevalence of PTSD in the general population was reported as 1.1% on average, ranging from 0.2 to 3.8%, in the World Mental Health survey^15^. This prevalence can be twice as high in women than men, higher in upper-middle-income countries, and most people affected have comorbid psychiatric disorders, particularly depression, anxiety, and substance abuse^16^. PTSS has a lifetime prevalence of 17%^17^. Brooks et al.^18^ pointed out in a review that posttraumatic stress symptoms occur in 28% to 34% of those in quarantine. In a recent meta-analysis about 20% of health care professionals reported PTSD^19^. Also, people who face the death of people from their network present a high risk for psychological distress^20^.

Health professionals carry a multitude of risk factors that may compromise mental health: they are often working in high-risk and long-hour conditions, they are generally in quarantine, and they commonly have sleep disturbances and comorbid mental health conditions^21,22^. Therefore, health professionals represent a vulnerable group to PTSS/PTSD in the COVID-19 pandemic, which is most unfortunate in a time when caring for their own health might be essential to mitigate population losses. To investigate how those who experienced a traumatic event are expressing PTSS and factors related to risk for higher symptomatology during the COVID-19 can inform how health professionals are facing their role in this crisis.

In this study, we investigated how many profiles of PTSS we could find for health professionals who experienced a traumatic event in their life using latent profile analysis. We then describe how latent profiles of PTSS symptoms could differ in terms of overall psychological distress, including regarding symptoms of depression and anxiety, and quality of life. Further, we investigated thoughts, experiences, and perceptions related to the COVID-19 pandemic in several areas as predictors of distinct PTSS profiles for health professionals.

## Methods

### Participants

The whole study protocol was evaluated and approved by the National Commission of Ethics in Research (CONEP) in Brazil (CAAE #: 30823620.6.0000.5149) and it follows international ethical regulations and standards. All participants were adults informed about the survey content, and all included gave an informed consent to participate. The Secretary of Education and Health Labor Management at the Ministry of Health in Brazil invited to participate in the survey all health professionals registered in their database by email. From the 1,971,464 health professionals with a tertiary degree (technical/professional or university/college degree) registered on Brazil’s National Health System, 223,866 (11.4%) accessed the consent form page but 2416 (1.1%) skipped the page. Data were collected from May 09th to June 30th, 2020, and 205,591 people (i.e., 92.8% of those answering the first page of the questionnaire) consented to participation. About 53,242 (25.9%) participants answered the outcome variable for this study, the Impact of Event Scale revised (IES-R) questionnaire, which is only appropriate to people who experienced a traumatic event in life. Participants with incomplete data in the IES-R (n = 3474) were excluded. 49,767 individuals were enrolled.

### Instruments

#### Impact of event scale-revised (IES-R)

The IES-R is a 22 question self-report scale designed to screen posttraumatic stress symptoms in adults or older people^23^. The IES-R has three subscales, intrusion (8 items), avoidance (8 items), and hyperarousal (6 items), and a total IES-R score. Items are rated on a 5-point scale ranging from 0 ("not at all") to 4 ("extremely"). Respondents rate their degree of distress during the past seven days after they have identified a specific stressful life event occurred to them. Scores are computed by averaging the items that make up the subscales, disregarding unanswered questions. The total score is the sum of the three subscales scores. The Brazilian version was culturally adapted and validated^24^.

#### Brief symptom inventory (BSI)^25^

The BSI is a 53-item instrument aiming to screen the intensity of psychological distress related to nine dimensions: somatization, obsession-compulsion, interpersonal sensitivity, depression, anxiety, hostility, phobic anxiety, paranoid ideation, and psychoticism. We computed the BSI’s Global Severity Index (GSI) composed of the mean score of all items answered for data analysis as a global measure of psychological distress. Since symptoms of depression and anxiety are frequently presented along with PTSS symptoms, depression (items 9, 16, 17, 18, 35, and 50), anxiety (items 1, 12, 19, 38, 45, and 49), and somatization (items 2, 7, 23, 29, 30, 33, and 37) scores were also computed to be compared among the latent profiles of the IER-S. The reliability of the Global Severity Index in the Brazilian version of BSI was ⍵ = 0.98; ⍵H = 0.95, which indicates high reliability^26^.

#### WhoQoL-BREF

The WHOQoL-BREF is a 26–item questionnaire measuring quality of life, i.e., individuals' perceptions of their position in life in the context of the culture and value systems in which they live and in relation to their goals, expectations, standards, and concerns, in four domains (physical, psychological, social relationships, and environment)^27–29^. The instrument is adequate when one aims to identify the impact of disease and impairment on daily activities and behavior. Items are self-scored on a 5-point scale ranging from 1 ("not at all") to 5 ("completely"). Domain scores are scaled in a positive direction (i.e., higher scores denote higher quality of life). The mean score of items within each domain is used to calculate the domain score.

#### Questions related to the COVID-19 outbreak

Sentences related to the COVID-19 outbreak were presented in a checkboxes format (checked = yes; non-checked = no). Participants were asked to select among all options those that applied to their experience in the last 14 days before responding to the questionnaire. We based most of the questionnaire about COVID-19 associated experiences on the same questions presented in the first study published on the psychological impacts of COVID-19 pandemic, which was done in China by Wang et al.^22^, adding questions we found appropriate for the Brazilian context at the time (i.e., April 2020). The structured questionnaire consisted of sentences that covered: (a) physical health status; (b) COVID-19 diagnosis and contact history, (c) perceptions and concerns related to COVID-19 pandemic, (d) precautionary measures against COVID-19 in the past 14 days, and (e) work and economic impressions related to the COVID-19 pandemic.

#### Brazilian economic classification criteria (CCEB)

For economic classification we used the Brazilian Economic Classification Criteria, a Brazilian instrument that provides evidence about the purchasing power and general situation of the households through questions about the possession of durable goods and the educational level of the head of the household. A subject score on CCEB can vary from 0 to 46 and it is classified in one of 6 classes with distinct average monthly income: A (R$ 25,554.33, 2.5% of the Brazilian population), B1 (R$ 11,279.14, 4.4% of the Brazilian population), B2 (R$ 5641.64, 16.5% of the Brazilian population), C1 (R$ 3085.48, 21.5% of the Brazilian population), C2 (R$ 1748.59, 26.8% of the Brazilian population), and DE (R$ 719.8, 28.3% of the Brazilian population).

### Procedures

The online survey was developed and collected through SurveyMonkey@. All researchers from this study and other collaborators tested the usability and technical functionality of the electronic questionnaire before fielding the questionnaire. There were 61 questions displayed through 13 pages in a fixed order. No incentives were offered for survey participation.

### Data analysis

To explore whether subpopulations within participants with the IES-R scale based on their scores, we carried out a latent profile analysis (LPA) using tidyLPA^30^ and profileR packages^31^ to conduct a person-centered analysis. LPA assumes that people can be typed with varying degrees of probabilities into categories that have different configural profiles of personal and/or environmental attributes. Usually, variable-centered methods allow us to identify the structure associated with different forms of psychopathology, however, their limitation lies precisely in identifying how many categories are suitable. In turn, person-centered methods, like latent profile analysis (LPA) are an alternative approach that allows the identification of relatively homogeneous sub-samples in a large population^32^, allowing a more individualized identification of individuals in relation to PTSS. To determine the number of profiles optimal for the IES-R data, we compared the goodness-of-fit indicators recommended by Nylund et al.: Bayesian Information Criterion (BIC), Integrated Completed Likelihood (ICL), and Bootstrapping (LRTS)^33^. Afterward, to perform an external validation of the IES-R profiles a Student's t-test was conducted to verify the extent to which the profiles differed in relation to psychological distress measures (BSI) and quality of life (WHOQoL-BREF).

Finally, multinomial logistic regression analysis was conducted to investigate the relationship between IES-R profiles, as an outcome, and COVID related sentences as predictors, using the forced entry method. Odds ratio (OR) and 95% confidence interval (CI) were calculated by the probability of the predictor indicated in differences observed among the IES-R profiles. These analyses were conducted using R software (R Core Team, 2020) and Rstudio (3.6.1)^34^, and we used p < 0.05 as significance level.

### Ethical evaluation

This study was approved by the National Commission of Ethics in Research (CONEP) on May 2nd, 2020 (CAAE #: 30823620.6.0000.5149) and complies with the Helsinki Declaration. All participants were informed that the survey would take about 25 min to be completed. The consent form was presented in the first page of the online form and only participants who consented to their participation were further enrolled. Participants should have 20 years or more and access to the Internet to enroll.

## Results

In the latent profile analysis (LPA), using the BIC index we compared various models with different numbers of profiles on the IES-R (Table 1) and we found three best models, with the best of them being the nine-profile model (BIC = − 249.538). Assessment of the ICL index showed that the best models would be the five-profile (ICL = − 276.310), three-profile (ICL = − 276,503.6861), and the four-profile (ICL = − 281,214.2) models. On its turn, comparison of the models according to the bootstrapping criterion indicated no differences between models with 1 up to 8 profiles. However, based on goodness-of-fit, optimal models included the two-profile and the three-profile and we opted for the two-profile model (LRTS = 2438.7972; p < 0.001) as the most appropriate for the IES-R data of the health professionals.

**Table 1:** The goodness of fit for the latent profile models.

Profile	AIC	BIC	SABIC	ICL	Entropy	LRTS	*P*
1	1.646.914	1.646.976	1.646.957	− 1.646.976	1.000	–	–
2	1.395.025	1.395.127	1.395.095	− 1.422.509	0.810	20.958.201	0.001
3	1.279.914	1.280.058	1.280.013	− 1.318.842	0.828	8.451.954	0.001
4	1.219.561	1.219.745	1.219.688	− 1.258.235	0.862	5.782.835	0.001
5	1.167.978	1.168.203	1.168.133	− 1.214.752	0.859	5.851.717	0.001
6	1.147.122	1.147.388	1.147.306	− 1.209.009	0.833	5.919.727	0.001
7	1.137.371	1.137.678	1.137.582	− 1.214.075	0.810	2.252.079	0.001
8	1.135.167	1.135.515	1.135.407	− 1.211.353	0.821	1.464.564	0.001
9	1.124.374	1.124.763	1.124.642	.1.197.454	0.837	3.123.052	0.001

*AIC* Akaike information criterion, *BIC* Bayesian information criterion, *SABIC* Sample size-adjusted Bayesian information criterion, *ICL* Integrated completed likelihood, *LRTS* Bootstrap sequential likelihood ratio test.

**Figure 1:**
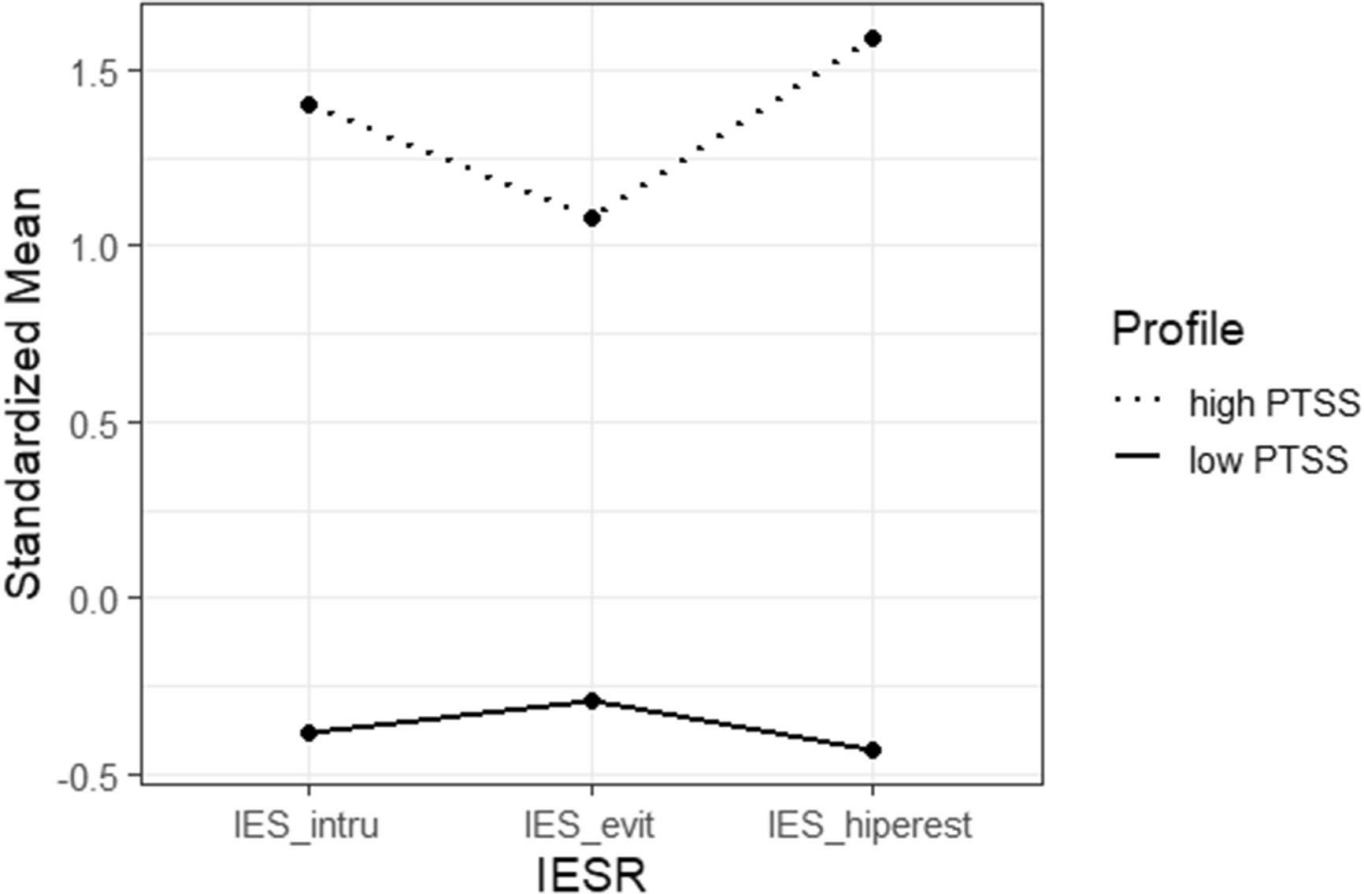
Latent profiles for IES-R scores. *IES_intru* Intrusion, *IES_evit* Avoidance, *IES_hiperest* hyperarousal.

Figure 1 shows the standardized z scores for each factor of IES-R (positive values indicate a greater presence of trauma related symptoms in the sample, while negative values indicate less symptoms) for each profile that we found. The first profile shows a high frequency of IES-R symptoms, so we referred to it as the traumatized profile (n = 10,401, 20.9%). The second pattern was characterized by low levels of trauma related symptoms, characterizing a not traumatized profile (n = 39,366, 79.1%).

Table 2 shows demographic information for the total sample and depending on the latent IES-R profile. We report on Table 3 descriptive statistics on IES-R, BSI, and WHOQoL-BREF dimensions for the overall sample and for each latent IES-R profile. To carry out the external validation of the two-profiles’ solution, we sought to differentiate them in terms of psychological distress (BSI measures), as well as quality of life (WHOQoL-BREF measures) using independent t tests (Supplementary Table 1).

**Table 2:** Demographic information for the total sample and for each IES-R’s profile: low PTSS and high PTSS.

Variable	Total Sample	Low PTSS	High PTSS
	(N = 49,767)	(N = 39,366)	(N = 10,401)
	M (SD)	M (SD)	M (SD)
Age	35.76 (9.47)	36.00 (9.60)	34.86 (8.93)
Socioeconomics (CCEB Brazil)	26.30 (8.27)	26.55 (8.27)	25.32 (8.19)

Sex	N (%)	N (%)	N (%)
Female	39,466 (79.64)	30,458 (77.71)	9008 (86.99)
Male	10,088 (20.35)	8741 (22.29)	1347 (13.01)
**Education**			
Doctorate degree	1616 (3.54)	1312 (3.62)	304 (3.25)
Master's	4159 (9.12)	3318 (9.15)	841 (9.01)
University/college degree	39,785 (87.32)	31,601 (87.22)	8184 (87.72)
**Marital status**			
Married/live together	22,587 (46.03)	18,273 (47.04)	4314 (42.19)
Divorced	4315 (8.79)	3323 (8.55)	992 (9.70)
Single	22,161 (45.16)	17,242 (44.39)	4919 (48.10)
**Occupation**			
Social Worker	1559 (7.34)	1215 (7.28)	344 (7.56)
Nursing assistant	334 (1.57)	258 (1.54)	76 (1.67)
Nurse	6020 (28.37)	4702 (28.20)	1318 (28.99)
Physiotherapist	5672 (26.73)	4507 (27.03)	1165 (25.63)
Nutritionist	4212 (19.85)	3275 (19.64)	937 (20.61)
Other	3419 (16.11)	2714 (16.28)	705 (15.51)
Missing	28,551	22,695	5856
**Ethnicity**			
Yellow (Asian/Oriental)	1610 (3.25)	1242 (3.17)	368 (3.56)
White	29,589 (59.82)	23,605 (60.32)	5984( 57.92)
Brown (parda)	15,085 (30.49)	11,784 (30.11)	3301 (31.95)
Black	3178 (6.42)	2500 (6.38)	678 (6.56)
**Economic class**			
A (highest)	1337 (2.68)	1120 (2.84)	217 (2.08)
B1	2992 (6.01)	2480 (6.30)	512 (4.92)
B2	13,453 (27.04)	10,927 (27.76)	2526 (24.30)
C1	15,601 (31.35)	12,386 (31.47)	3215 (30.92)
C2	11,058 (22.22)	8482 (21.55)	2576 (24.78)
D–E (lowest)	5308 (10.67)	3959 (10.06)	1349 (12.97)
	Yes/no	Yes/no	Yes/no
Trauma stress	13,806 (27.74)/35,961 (72.25)	10,904 (27.69)/28,462 (72.30)	2902 (27.90)/7499 (72.09)
Trauma direct exposure	7294 (14.65)/42,473 (85.34)	5758 (14.62)/33,608 (85.37)	1536 (14.76)/8865 (85.23)
Trauma witness	4198 (8.43)/45,569 (91.56)	3289 (8.35)/36.077 (91.64)	909 (8.74)/9492 (91.26)
Trauma learned	4887 (9.82)/44,880 (90.18)	3852 (9.78)/35,514 (90.21)	1035 (9.95)/9366 (90.04)
Trauma indirect exposure	1215 (2.44)/48,552 (97.55)	961 (2.44)/38,405 (97.55)	254 (2.44)/10,147 (97.55)

**Table 3:** Descriptive statistics for IES-R, BSI, and WHOQoL-BREF dimensions for the total sample and for each IES-R’s profile: low-PTSS and high-PTSS.

Variable	Total sample (n = 49,767)			Low-PTSS (n = 39,366)			High-PTSS (n = 10,401)		
	M	SD	Min–Max	M	SD	Min–Max	M	SD	Min–Max
**IES-R**									
Intrusion	0.905	0.916	0–4	0.549	0.540	0–3.87	2.252	0.782	0–4
Evitation	1.087	0.933	0–4	0.813	0.770	0–4	2.126	0.744	0–4
Hiperestimulation	0.815	0.916	0–4	0.418	0.440	0–2.16	2.315	0.659	0.66–4
Total	2.807	2.575	0–12	1.78	1.55	0–9.91	6.693	1.88	2.0–9.91
**BSI**									
GSI	0.792	0.65	0–4	0.631	0.523	0–4	1.403	0.716	0–4
Somatization	0.484	0.601	0–4	0.360	0.477	0–4	0.952	0.767	0–4
Depression	0.943	0.856	0–4	0.764	0.729	0–4	1.621	0.954	0–4
Anxiety	0.985	0.821	0–4	0.795	0.683	0–4	1.702	0.898	0–4
**WhoQol-BREF**									
Physical	3.685	0.688	1–5	3.820	0.627	1.14–5	3.175	0.671	1–5
Psychological	3.486	0.733	1–5	3.628	0.670	1–5	2.947	0.707	1–5
Social	3.361	0.844	1–5	3.483	0.801	1–5	2.903	0.847	1–5
Environmental	3.275	0.638	1–5	3.364	0.605	1–5	2.938	0.651	1–5

**Figure 2:**
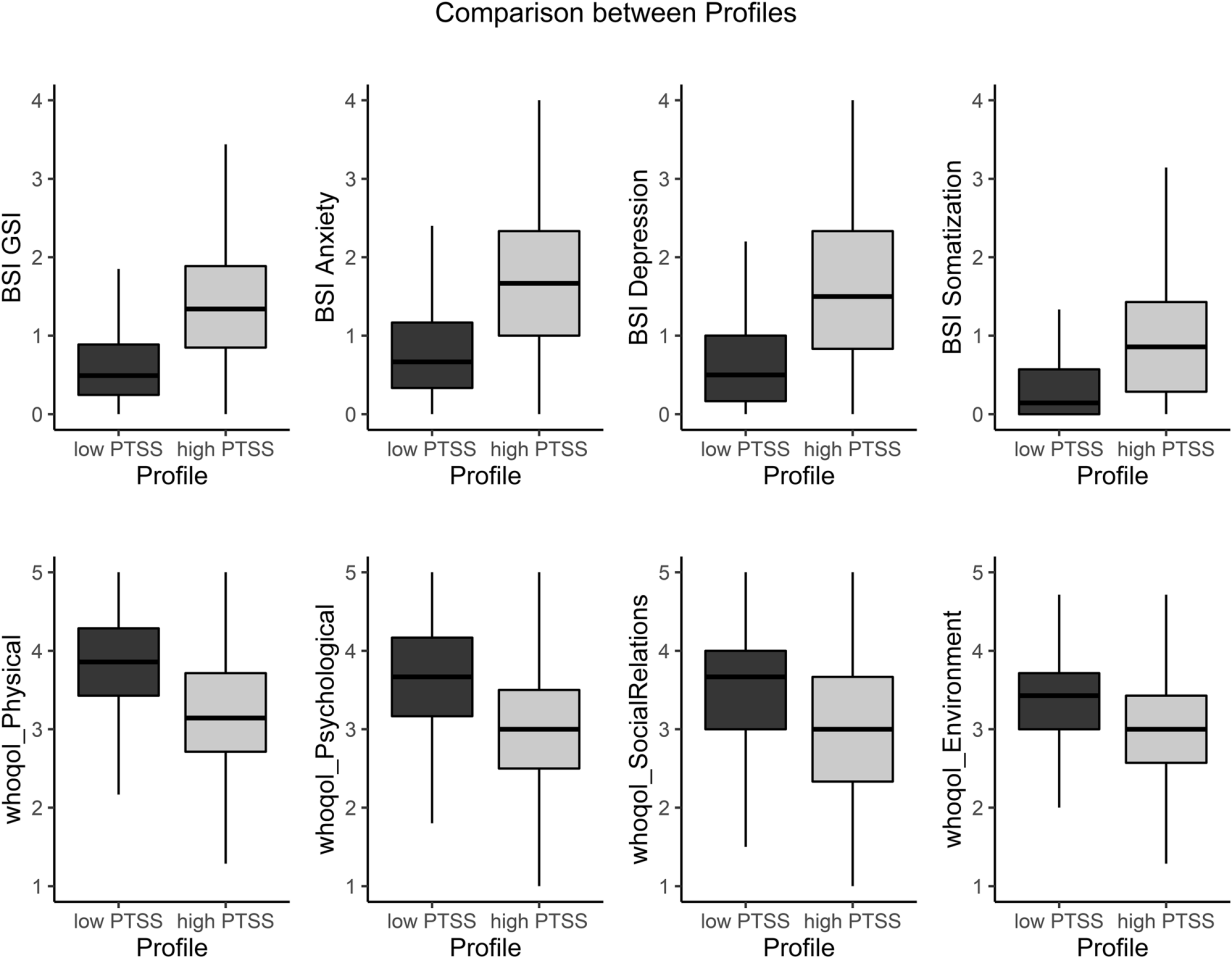
Boxplot diagrams comparing IES-R’ profiles (traumatized vs. not traumatized) in relation to psychological distress (BSI measures) and quality of life (WHOQoL-BREF).

As we can see in Fig. 2, results indicate significant differences between the low-PTSS and high-PTSS profiles regarding psychological distress with the high-PTSS group showing much higher scores than the low-PTSS group (GSI [t(13,462) = − 102.93, p < 0.0001; Cohen’s d = 0.66], anxiety [t(13,732) = − 95.872, p < 0.0001; Cohen’s d = 0.63], depression [t(13,761) = − 85.211, p < 0.0001; Cohen’s d = 0.58], and somatization [t(12,592) = − 74.987, p < 0.0001; Cohen’s d = 0.55]). For quality of life, there was also significant differences between the high-PTSS and low-PTSS groups, with lower mean scores for the high-PTSS group in the physical ([t(15,691) = 88.407, p < 0.0001; Cohen’d = 0.57]), psychological ([t(15,647) = 62.784, p < 0.0001; Cohen’d = 0.44]), social ([t(15,466) = 60.24, p < 0.0001; Cohen’d = 0.43]), and environment ([t(15,466) = 60.24, p < 0.0001; Cohen’d = 0.43]) domains. Together, these results indicate that identifying latent profiles of the IES-R might be a useful way to differentiate participants in relation to levels of PTSS and its predictors.

Among predictors of participants’ profiles depending on their IER-S profile, i.e., low-PTSS and high-PTSS, were found as characteristics of risk for the high-PTSS (see Table 3): female sex, lower education, divorced and single participants, participants from lower economic classes, participants more frequently reporting chills, headache, myalgia, breathing difficulty, dizziness, sore throat, chest pressure, and bluish lips or face as physical symptoms; health professionals recently hospitalized or without a COVID-19 diagnosis; people who were afraid of transmitting the virus that causes COVID-19 to a family member, or lost a family member or close friend due to COVID-19; people who were not working or studying from home (home office), who did not kept moving outdoors (leaving home) for work as usual; participants who did needed to leave home for work but were afraid of COVID-19; people who did not declare taking preventive measures such as staying at least 1 m apart from people when out of home, who do not sanitizes hands with alcohol gel (70% ethyl alcohol) or wash hands for at least 20 s, whenever possible when out of home, or do not avoid touching the face as much as possible, especially when out of home, were more frequently on the traumatized group. On the other hand, professionals who were careful to not touch anything after coughing or seeing someone coughing nearby were more likely to present the low-PTSS profile. People on the high-PTSS profile more likely felt that the quality of the relationship between people at home had worsened, felt less productive at work, and received basic assistance from friends or neighbors. Risk factors also included do not trust COVID-19 information from traditional TV and radio, confused about how to protect themselves from COVID-19, very afraid of not being able to deal with present or yet to come financial difficulties, does not think that COVID-19 is a mild infection, thinking that COVID-19 is a severe infection, does not believe that economic struggles related to social distancing measures will be overcome soon (up to 2 years), and perception that social distancing increased violence around me (at home/neighborhood).

## Discussion

We found two latent profiles of PTSS in health professionals who reported having ever lived a traumatic experience. One profile consisted of health professionals with lower PTSS, termed here as the low-PTSS profile, and a second profile with participants with high PTSS (intrusion, evitation, and hiperestimulation), called the high-PTSS profile. The high-PTSS profile demonstrated worse psychological scores (global psychological distress, somatization, depression, and anxiety) and worse quality of life (physical, psychological, social, and environmental) with moderate magnitudes. The prevalence of PTSD was about 20% among health care professionals in a meta-analysis data, however it ranges from 7 to 70% and the risk features need to be understood to have a better picture of the reasons related to having trauma^19,35^.

Among possible risk factors for the  high-PTSS, we investigated several sociodemographic factors and COVID-19 experiences, thoughts, and perceptions. Small but significant predictors of the PTSS profile included the female sex, having lower education, divorced and single participants, participants from lower economic classes, participants more frequently reporting physical symptoms related to the COVID-19 infection; having been hospitalized or not having had a COVID-19 diagnosis; fear of transmitting the virus, losing a family member or close friend due to COVID-19; to work out of home afraid of COVID-19; not taking preventive measures but caring for fomite transmission; perception that the relationship between people at home had worsened, feeling less productive at work, and receiving basic assistance from friends or neighbors. Being confused about how to protect themselves from COVID-19, thinking that COVID-19 is not a mild infection, fear of prolonged economic struggles, and perception of increased violence nearby were also associated with the traumatized profile.

We expect a mental health compromise due to disruption of the state of well-being^36^. The pandemic condition disrupts the well-being and life in society, but for some, that period also meant excessive hours of work, though decisions and health risk for them and their close ones. Health professionals have been the essential professionals to overcome the pandemic, and a risk group for stress disorders by their occupational exposures. As with any extreme condition, most of us will not feel traumatized by the pandemic experience, but there are some risk factors that might increase the risk to carry longer effects of this pandemic. We need to understand who is more vulnerable to have mental health compromised to mitigate the effect while living the process and the problems related to the COVID-19 pandemic^37^.

A group emerged as at riskier condition to have PTSS: the individuals with high scores of depression, anxiety, and low quality of life in any domain. This group was also identified as a riskier to have any mental disorder. However, we do not have a baseline condition to know if these persons felt more the impact of the pandemic or if they had previous mental health disorders that worsened due the pandemic stress. Previous findings suggested that the individuals with past-psychiatric disorders or psychological adjustment might have a higher risk to develop posttraumatic disorders^14,38–40^. However, the warning that individuals with psychiatric disorders should be a target population to be followed and cared for in the pandemic has been repeated in the literature on mental health impact^14,40^.

Some psychiatric disorders are more prevalent in women^41,42^. In our study, more women are informing more about posttraumatic symptoms, as the previous study with Chinese, and Italian samples, or in health professionals^14,40,43^. They report symptoms in three re-experiencing, negative alterations in cognition or mood, and hyper-arousal domains. Like here, even at the very beginning of the pandemic, the PTSS was present a month after the COVID-19 in Wuhan, reaching 7% already^13^. Experimental studies found that women were more reactive than men in neural networks associated with fear and arousal responses^44^, and greater differential conditioned skin conductance responses to aversive stimuli^45^. The hyper-activation of neural fear processing networks can explain gender differences in the PTSS prevalence^46^. The possibility that women present more symptoms after traumatic events, suggests that there is a need to better understand this gender-relationship in order to identify targets to intervene^47^.

Most of the identified risk features in our study are classically related to PTSS and PTSD in other traumatic events^48,49^. Some demographic features slightly increased the risk of feeling traumatized: the lonely, lower socioeconomic status, the less educated, the ones who still have to be physically at work, the ones who are fearing to struggle economically. In the personal field, an interesting point emerged as a risk: the home relationship quality. From a previous longitudinal study after an earthquake, there is evidence suggesting that symptoms of PTSS influence the partner relationship, which solves after some time after the event^50^. Thus, we have the limitations inherent from a cross-sectional study; only longitudinally we will better understand the dynamic of home relationships and the COVID-19 pandemic for health professionals.

Some particular risk factors emerged from this pandemic and its characteristics. People are now alert about cold symptoms, with many of them being pointed out as risky features to PTSS. The recognition of signs of worst severity soon disseminated the fear of having them. So, the presence of signs of hypoxia and thoracic pain became the highest known risks to develop trauma. Directly related to this pandemic was identifying the loss of someone close as a risk factor for trauma, which amplifies the negative psychological effects^20,40^. Besides the particularities of the pandemic condition, the fear to transmit to family mimics the risks identified in individuals who had a trauma related to motor vehicle accidents. The latter also shows that the presence of someone in the car during the accident increased the risk of developing trauma, offering the same pattern of the actual findings^51^. Spare the close ones might consist in a strategy to promote well-being to the healthcare workers, such as what is happening with the availability of hotels and accommodations to be isolated during the contact with infectious risk.

In the beginning of the pandemic, more traumatized health professionals report fear of working and feel confused about how to protect themselves. Their responses sound natural when facing a new pandemic with an unknown disorder; we expect that the increase of the knowledge of the disease and the improvement of preparedness sense mitigate this response. During the pandemic, the government provided online lessons about the disease and its management for health professionals. Thus, our measures might not reflect this training and the cumulative knowledge since data was collected at the beginning of the outbreak in Brazil. However, we are following this population longitudinally, the effect of this learning might be better understood in the subsequent waves.

We live in a middle-income country with periodic times of economic crises and related consequences. Some risk factors might have emerged by the fear and the remembering of this past. In the pandemic, our Brazilian population reports fear of the economic struggle. This might be important to reinforce the policy to buffer the economic suffering and to minimize mental health impact, potentially saving money in the long run.

Previous meta-analysis data on PTSS observed that the most critical research target was not the sociodemographic information but the severity of PTSS, history of previous traumas, psychological adjustment, family history of psychopathology, and experiences related to the trauma^39,52^. IES-R has good accuracy for chronic PTSD identification^53^. However, our study was designed to screen mental health status at the beginning of the pandemic. We have no diagnostic information, and more studies are necessary to explore further the impact of acute and past histories in the traumatic individual experience in this condition. The most important information was the risk features identified and conferring risk to feel traumatized for a while. We are aware that large online surveys have the potential for self-reporting bias; however, there is extensive data widely distributed for all registered professionals. We also used a robust statistical strategy to identify groups based on a latent approach that provides rigorous statistical tests to assess model fit and offer an objective criterion for how many clusters are supported by the data.

In conclusion, the large surveys reaching many citizens might be a good source of information about disorder cases irrespective of service use in pandemics. Pandemic is related to an increase of the PTSS depending on the individual risks; for most, fortunately, our results show that individuals were classified in a profile without related trauma. The fact that a pandemic is happening almost simultaneously in the whole world, calls us for action and warns us that we need to prepare ourselves for better short-term responses and this study helps in the identification of potential targets.

## Supplementary Information

Supplementary Information.

## Data Availability

The datasets generated during and/or analysed during the current study are available from the last author on reasonable request.

## References

[1] Xiong J (2020). Impact of COVID-19 pandemic on mental health in the general population: A systematic review. J. Affect. Disord..

[2] Kronenberg ME (2010). Children of Katrina: Lessons learned about postdisaster symptoms and recovery patterns. Child Dev..

[3] Makwana N (2019). Disaster and its impact on mental health: A narrative review. J. Family Med. Prim. Care.

[4] Profiles, E.-D. D. *The OFDA/CRED International Disaster Database*. (2020).

[5] Perry RW, Rodríguez H, Donner W, Trainor JE (2018). Defining disaster: An evolving concept. Handbook of Disaster Research.

[6] Abdallah CG (2019). Reduced salience and enhanced central executive connectivity following PTSD treatment. Chronic Stress.

[7] Guan W-J (2020). Clinical characteristics of coronavirus disease 2019 in China. N. Engl. J. Med..

[8] Mavergames, C. & Elliot, J. Living systematic reviews: Towards real-time evidence for health-care decision-making. *BMJ Best Pract. *(2019).

[9] Abdallah CG (2019). The neurobiology and pharmacotherapy of posttraumatic stress disorder. Annu. Rev. Pharmacol. Toxicol..

[10] Vermetten E, Bremner JD (2002). Circuits and systems in stress. II. Applications to neurobiology and treatment in posttraumatic stress disorder. Depress. Anxiety.

[11] Choumert-Nkolo J, Phélinas P (2019). Natural disasters, land and labour. Eur. Rev. Agric. Econ..

[12] Islam MR (2018). Climate change, natural disasters and socioeconomic livelihood vulnerabilities: Migration decision among the Char land people in Bangladesh. Soc. Indic. Res..

[13] Liu N (2020). Prevalence and predictors of PTSS during COVID-19 outbreak in China hardest-hit areas: Gender differences matter. Psychiatry Res..

[14] Qiu J (2020). A nationwide survey of psychological distress among Chinese people in the COVID-19 epidemic: Implications and policy recommendations. Gen. Psychiatry.

[15] Karam EG (2014). Cumulative traumas and risk thresholds: 12-month PTSD in the World Mental Health (WMH) surveys. Depress. Anxiety.

[16] Yuan K (2021). Prevalence of posttraumatic stress disorder after infectious disease pandemics in the twenty-first century, including COVID-19: A meta-analysis and systematic review. Mol. Psychiatry.

[17] Zhou Y (2021). The prevalence of PTSS under the influence of public health emergencies in last two decades: A systematic review and meta-analysis. Clin. Psychol. Rev..

[18] Brooks SK, Webster RK, Smith LE, Woodland L (2020). The psychological impact of quarantine and how to reduce it: rapid review of the evidence. Lancet.

[19] Li Y, Scherer N, Felix L, Kuper H (2021). Prevalence of depression, anxiety and post-traumatic stress disorder in health care workers during the COVID-19 pandemic: A systematic review and meta-analysis. PLoS ONE.

[20] Joaquim RM (2021). Bereavement and psychological distress during COVID-19 pandemics: The impact of death experience on mental health. Curr. Res. Behav. Sci..

[21] Imo UO (2017). Burnout and psychiatric morbidity among doctors in the UK: A systematic literature review of prevalence and associated factors. J. Psychol. Bull.

[22] Wang C (2020). A longitudinal study on the mental health of general population during the COVID-19 epidemic in China. Brain Behav. Immun..

[23] Weiss DS (2007). The impact of event scale: Revised. Cross-Cult. Assess. Psychol. Trauma PTSD.

[24] Caiuby AVS, Lacerda SS, Quintana MI, Torii TS, Andreoli SB (2012). Adaptação transcultural da versão brasileira da Escala do Impacto do Evento: Revisada (IES-R). Cad. Saúde Publ..

[25] Derogatis LR, Melisaratos N (1983). The brief symptom inventory: An introductory report. Psychol. Med..

[26] Serpa ALO (2021). Brief symptom inventory: Reporting Brazilian populational parameters during COVID-19 pandemics. Archives.

[27] Skevington SM, Bradshaw J, Saxena S (1999). Selecting national items for the WHOQOL: Conceptual and psychometric considerations. Soc. Sci. Med..

[28] THE Whoqol GROUP (1998). Development of the World Health Organization WHOQOL-BREF quality of life assessment. Psychol. Med..

[29] da Rocha NS, de Fleck MP (2009). Validity of the Brazilian version of WHOQOL-BREF in depressed patients using Rasch modelling. Rev. Saude Publ..

[30] Rosenberg, J. M., Schmidt, J. A., Beymer, P. N. & Steingut, R. R. Interface to mclust to easily carry out latent profile analysis. *Stat. Softw. R*. (2018).

[31] Desjardins J (2005). L’analyse de régression logistique. Tutor. Quant. Methods Psychol..

[32] Olino TM, Klein DN, Farmer RF, Seeley JR, Lewinsohn PM (2012). Examination of the structure of psychopathology using latent class analysis. Compr. Psychiatry.

[33] Nylund KL, Asparouhov T, Muthén BO (2007). Deciding on the number of classes in latent class analysis and growth mixture modeling: A Monte Carlo simulation study. Struct. Equ. Modeling.

[34] Team, R. *RStudio: Integrated Development for R. RStudio Inc. (2015). Acknowledgements This work was supported by Fundação para a Ciência e Tecnologia (FCT)* (2020).

[35] Benfante A, Di Tella M, Romeo A, Castelli L (2020). Traumatic stress in healthcare workers during COVID-19 pandemic: A review of the immediate impact. Front. Psychol..

[36] McKinzie AE (2018). In their own words: Disaster and emotion, suffering, and mental health. Int. J. Qual. Stud. Health Well Being.

[37] de Souza Costa D (2021). Predictors of psychological distress and prevalence of self-reported mental disorders across healthcare professionals and the general population during the COVID-19 outbreak in Brazil. PsyArXiv.

[38] Kilpatrick DG (2003). Violence and risk of PTSD, major depression, substance abuse/dependence, and comorbidity: Results from the National Survey of Adolescents. J. Consult. Clin. Psychol..

[39] Ozer EJ, Best SR, Lipsey TL, Weiss DS (2003). Predictors of posttraumatic stress disorder and symptoms in adults: A meta-analysis. Psychol. Bull..

[40] Rossi R (2020). COVID-19 pandemic and lockdown measures impact on mental health among the general population in Italy. Front. Psychiatry.

[41] McLean CP, Anderson ER (2009). Brave men and timid women? A review of the gender differences in fear and anxiety. Clin. Psychol. Rev..

[42] Kendler KS, Thornton LM, Prescott CA (2001). Gender differences in the rates of exposure to stressful life events and sensitivity to their depressogenic effects. Am. J. Psychiatry.

[43] Di Tella M, Romeo A, Benfante A, Castelli L (2020). Mental health of healthcare workers during the COVID-19 pandemic in Italy. Authorea.

[44] Felmingham K (2010). Neural responses to masked fear faces: Sex differences and trauma exposure in posttraumatic stress disorder. J. Abnorm. Psychol..

[45] Inslicht SS (2013). Sex differences in fear conditioning in posttraumatic stress disorder. J. Psychiatr. Res..

[46] Olff M, Langeland W, Draijer N, Gersons BPR (2007). Gender differences in posttraumatic stress disorder. Psychol. Bull..

[47] d’Ettorre G (2021). Post-traumatic stress symptoms in healthcare workers dealing with the COVID-19 pandemic: A systematic review. Int. J. Environ. Res. Public Health.

[48] Brewin CR, Andrews B, Valentine JD (2000). Meta-analysis of risk factors for posttraumatic stress disorder in trauma-exposed adults. J. Consult. Clin. Psychol..

[49] Silver RC, Holman EA, McIntosh DN, Poulin M, Gil-Rivas V (2002). Nationwide longitudinal study of psychological responses to September 11. JAMA.

[50] Marshall EM, Kuijer RG, Simpson JA, Szepsenwol O (2017). Standing on shaky ground? Dyadic and longitudinal associations between posttraumatic stress and relationship quality postearthquake. J. Fam. Psychol..

[51] Dougall AL, Ursano RJ, Posluszny DM, Fullerton CS, Baum A (2001). Predictors of posttraumatic stress among victims of motor vehicle accidents. Psychosom. Med..

[52] Shalev AY (2019). Estimating the risk of PTSD in recent trauma survivors: Results of the international consortium to predict PTSD (ICPP). World Psychiatry.

[53] Mouthaan J, Sijbrandij M, Reitsma JB, Gersons BPR, Olff M (2014). Comparing screening instruments to predict posttraumatic stress disorder. PLoS ONE.

