# Supplementary material for: Increased risk of health professionals to feel traumatized during the COVID-19 pandemic
Source: Sci Rep. 2021 Sep 14;11:18286. doi: 10.1038/s41598-021-97783-6 (PMC8440540; doi:10.1038/s41598-021-97783-6)
Supplement: Supplementary file 1 — Supplementary Information. [file 41598_2021_97783_MOESM1_ESM.docx]

**Increased risk of health professionals to feel traumatized during the COVID-19 pandemic**

André Luiz de Carvalho Braule Pinto^a,b,^ Alexandre Luiz de Oliveira Serpa^a,d,^ Jonas Jardim de Paula^a,b,c^, Danielle de Souza Costa^a,b,^ Kelly Robis^a,b^, Alexandre Paim Diaz^a,e,^ Rui Mateus Joaquim^a,b,^ Antônio Geraldo da Silva^a,f,g^, Débora Marques de Miranda^a,i^, Leandro Fernandes Malloy-Diniz^a,h^

***Supplementary material***

*Table S1. Results for multinomial logistic regression analysis with the latent profiles (subgroups) of the IES-R as outcome and Odds Ratio for each COVID-19 related event or perception*

| ***Coefficient:*** | ***B*** | ***SE*** | ***Pr(>\|z\|)*** | ***OR*** | ***95% CI for OR*** | |
| --- | --- | --- | --- | --- | --- | --- |
|  |  |  |  |  | ***Lower*** | ***Upper*** |
| *(Intercept) **** | *-1.846* | *0.139* | *< 0.001* | *0.16* | *0.12* | *0.21* |
| *Sex [Masculine] **** | *-0.475* | *0.035* | *< 0.001* | *0.62* | *0.58* | *0.67* |
| *Age [in years]* | *0.001* | *0.001* | *0.371* | *1.00* | *1.00* | *1.00* |
| *Education [Master’s degree]* | *-0.112* | *0.080* | *0.161* | *0.89* | *0.76* | *1.05* |
| *Education [Undergraduate] ** | *-0.150* | *0.072* | *0.037* | *0.86* | *0.75* | *0.99* |
| *Marital status [Divorced(a)] **** | *0.234* | *0.046* | *< 0.001* | *1.26* | *1.15* | *1.38* |
| *Marital status [Single(a)] **** | *0.121* | *0.028* | *< 0.001* | *1.13* | *1.07* | *1.19* |
| *CCEB Class [B1]* | *-0.032* | *0.094* | *0.725* | *0.97* | *0.81* | *1.17* |
| *CCEB Class [B2]* | *0.023* | *0.082* | *0.778* | *1.02* | *0.87* | *1.21* |
| *CCEB Class [C1]* | *0.095* | *0.082* | *0.244* | *1.10* | *0.94* | *1.30* |
| *CCEB Class [C2] *** | *0.219* | *0.083* | *0.008* | *1.25* | *1.06* | *1.47* |
| *CCEB Class [D-E] **** | *0.322* | *0.089* | *< 0.001* | *1.38* | *1.16* | *1.65* |
| *Persistent fever* | *-0.066* | *0.079* | *0.397* | *0.94* | *0.80* | *1.09* |
| *Chills*** | *0.147* | *0.048* | *0.002* | *1.16* | *1.05* | *1.27* |
| *Headache**** | *0.199* | *0.029* | *< 0.001* | *1.22* | *1.15* | *1.29* |
| *Myalgia**** | *0.152* | *0.029* | *< 0.001* | *1.16* | *1.10* | *1.23* |
| *Cough* | *0.005* | *0.036* | *0.876* | *1.01* | *0.94* | *1.08* |
| *Breathing difficulty**** | *0.199* | *0.046* | *< 0.001* | *1.22* | *1.11* | *1.34* |
| *Dizziness**** | *0.300* | *0.037* | *< 0.001* | *1.35* | *1.26* | *1.45* |
| *Coryza* | *0.022* | *0.030* | *0.460* | *1.02* | *0.96* | *1.08* |
| *Sore throat**** | *0.152* | *0.034* | *< 0.001* | *1.16* | *1.09* | *1.25* |
| *Chest pressure**** | *0.406* | *0.038* | *< 0.001* | *1.50* | *1.39* | *1.62* |
| *Bluish lips or face**** | *0.625* | *0.170* | *< 0.001* | *1.87* | *1.34* | *2.61* |
| *Consultation with a medical doctor**** | *0.158* | *0.044* | *< 0.001* | *1.17* | *1.07* | *1.28* |
| *Hospitalization** | *0.211* | *0.208* | *0.310* | *1.24* | *0.82* | *1.85* |
| *Tested for COVID-19* | *-0.135* | *0.066* | *0.04* | *0.87* | *0.77* | *0.99* |
| *Diagnosed with COVID-19*** | *-0.392* | *0.120* | *0.001* | *0.68* | *0.53* | *0.85* |
| *Put in quarantine by a healthcare professional* | *-0.048* | *0.072* | *0.505* | *0.95* | *0.83* | *1.10* |
| *Direct contact with a person with COVID-19* | *0.051* | *0.040* | *0.202* | *1.05* | *0.97* | *1.14* |
| *Family member or close friend with COVID-19* | *0.063* | *0.034* | *0.070* | *1.07* | *0.99* | *1.14* |
| *Afraid of transmitting the virus that causes COVID-19 to a family member**** | *0.160* | *0.028* | *< 0.001* | *1.17* | *1.11* | *1.24* |
| *Lost a family member or close friend due to COVID-19**** | *0.218* | *0.048* | *< 0.001* | *1.24* | *1.13* | *1.37* |
| *Working or studying from home (home office) *** | *-0.095* | *0.034* | *0.005* | *0.91* | *0.85* | *0.97* |
| *Already worked from home before COVID-19 pandemic* | *0.059* | *0.085* | *0.492* | *1.06* | *0.89* | *1.25* |
| *Working under reduced hours or taking turns with coworkers**** | *-0.205* | *0.032* | *< 0.001* | *0.81* | *0.76* | *0.87* |
| *Waiting social distancing rules' suspension to go back to working or studying* | *0.037* | *0.050* | *0.456* | *1.04* | *0.94* | *1.14* |
| *Kept moving outdoors (leaving home) for work as usual*** | *-0.095* | *0.032* | *0.003* | *0.91* | *0.85* | *0.97* |
| *Need to leave home for work but is afraid of COVID-19**** | *0.340* | *0.029* | *< 0.001* | *1.41* | *1.33* | *1.49* |
| *Work directly in the healthcare workforce for COVID-19* | *-0.077* | *0.079* | *0.332* | *0.93* | *0.79* | *1.08* |
| *Stays at least 1 meter apart from people when out of home**** | *-0.105* | *0.031* | *< 0.001* | *0.90* | *0.85* | *0.96* |
| *Sanitizes hands with alcohol gel (70% ethyl alcohol) or wash hands for at least 20 seconds, whenever possible when out of home*** | *-0.105* | *0.039* | *0.008* | *0.90* | *0.83* | *0.97* |
| *Avoids touching the face as much as possible, especially when out of home**** | *-0.148* | *0.037* | *< 0.001* | *0.86* | *0.80* | *0.93* |
| *Is very careful to not touch anything after coughing or seeing someone coughing nearby*** | *0.082* | *0.031* | *0.008* | *1.09* | *1.02* | *1.15* |
| *Only leaves home when extremely necessary and wearing a face covering* | *-0.001* | *0.032* | *0.950* | *1.00* | *0.94* | *1.06* |
| *Kept moving outdoors (leaving home) as usual, like before the COVID-19 outbreak* | *0.047* | *0.104* | *0.652* | *1.05* | *0.85* | *1.28* |
| *Started using video calling apps/softwares very often* | *-0.033* | *0.028* | *0.240* | *0.97* | *0.91* | *1.02* |
| *Feels that the quality of the relationship between people at home has improved* | *-0.041* | *0.029* | *0.157* | *0.96* | *0.91* | *1.02* |
| *Feels that the quality of the relationship between people at home has worsened**** | *0.423* | *0.035* | *< 0.001* | *1.53* | *1.43* | *1.64* |
| *Feels more productive at work* | *0.004* | *0.044* | *0.924* | *1.00* | *0.92* | *1.09* |
| *Feels less productive at work**** | *0.285* | *0.028* | *< 0.001* | *1.33* | *1.26* | *1.41* |
| *Received financial assistance from the government* | *0.039* | *0.040* | *0.327* | *1.04* | *0.96* | *1.13* |
| *Received basic assistance from friends or neighbors**** | *0.173* | *0.052* | *< 0.001* | *1.19* | *1.07* | *1.32* |
| *Received basic assistance from volunteers* | *0.127* | *0.126* | *0.313* | *1.14* | *0.88* | *1.45* |
| *Went to a hospital or clinic but was denied healthcare due to the absence of a vacancy (health system overload)* | *-0.118* | *0.170* | *0.485* | *0.89* | *0.63* | *1.24* |
| *Agrees with social distancing rules* | *-0.074* | *0.041* | *0.071* | *0.93* | *0.86* | *1.01* |
| *Does not agree with social distancing rules* | *-0.107* | *0.057* | *0.06* | *0.90* | *0.80* | *1.00* |
| *Trusts COVID-19 information from traditional TV and radio*** | *-0.089* | *0.027* | *0.001* | *0.91* | *0.87* | *0.97* |
| *Trusts COVID-19 information from the Internet (social medias)* | *-0.017* | *0.042* | *0.683* | *0.98* | *0.90* | *1.07* |
| *Confused about how to protect yourself from COVID-19**** | *0.325* | *0.068* | *< 0.001* | *1.39* | *1.21* | *1.58* |
| *Very afraid of not being able to deal with present or yet to come financial difficulties**** | *0.413* | *0.026* | *< 0.001* | *1.51* | *1.44* | *1.59* |
| *Has donated to help fight COVID-19* | *-0.032* | *0.028* | *0.257* | *0.97* | *0.92* | *1.02* |
| *Has volunteered to help fight COVID-1* | *0.039* | *0.040* | *0.334* | *1.04* | *0.96* | *1.13* |
| *Thinks that COVID-19 is a mild infection** | *-0.201* | *0.082* | *0.014* | *0.82* | *0.69* | *0.96* |
| *Thinks that COVID-19 is a severe infection** | *0.064* | *0.032* | *0.046* | *1.07* | *1.00* | *1.14* |
| *Believes that economic struggles related to social distancing* measures will be overcome soon* | *-0.108* | *0.042* | *0.011* | *0.90* | *0.83* | *0.98* |
| *Believes that economic struggles related to social distancing measures will last longer* | *-0.069* | *0.041* | *0.091* | *0.93* | *0.86* | *1.01* |
| *Social distancing increased violence around me (at home/neighborhood) **** | *0.404* | *0.040* | *< 0.001* | *1.50* | *1.38* | *1.62* |

*Note: CCEB = Brazilian Economic Classification Criterion (classes are presented from higher to lower in alphabetical order); reference subgroup was high PTSS profile.*

**p<0.05*

***p<0.01*

****p<0.001*
